# Longer Rodent Bioassay: Huff et al. Respond

**DOI:** 10.1289/ehp.11964R

**Published:** 2008-12

**Authors:** James Huff, Michael F. Jacobson, Devra Lee Davis

**Affiliations:** National Institute of Environmental Health Sciences, National Institutes of Health Department of Health and Human Services, Research Triangle Park, North Carolina, E-mail: huff1@niehs.nih.gov; Center for Science in the Public Interest, Washington, DC; Center for Environmental Oncology, University of Pittsburgh Cancer Institute, Department of Epidemiology, Graduate School of Public Health, Pittsburgh, Pennsylvania

We appreciate and sympathize with Manuppello and Willett’s concerns about the inappropriate use of animals in research. Significant advances in animal protection have been secured in the United States through standards that have been promulgated, and we hope that further progress will be made.

Drugs are developed today often using methods that evaluate impacts *in vitro* and take advantage of innovations in three-dimensional structure-activity modeling. Still, because *in vitro* and theoretical methods are imperfect and because relying on them alone could result in considerable harm to humans, drugs and other chemicals are studied—and are required to be studied—in animals before being tested on humans or introduced into the human environment.

Epidemiologic studies, high-throughput *in vitro* methods, and computational toxicology would be preferable to animal research if they provided sensitive, accurate measures of human risk. Unfortunately, epidemiology studies are insufficiently sensitive, *in vitro* methods are insufficiently predictive, and computational toxicology is insufficiently developed. Ideally, government would provide much greater funding to further develop those and other technologies to expedite less expensive and more accurate means of risk assessments and reduce the need for animal bioassays.

The fact that positive animal bioassays have not always been mirrored in positive human findings provides an ethical public policy challenge. If we must wait for human evidence of cancer before acting to prevent future cases, then we are conducting experiments on unwitting subjects without controls—especially if animal evidence already indicated a risk.

Current standards for concluding that an agent is a human carcinogen require statistically significant proof of sufficient numbers of cases of cancer in humans with measured or estimated exposures. Human cancers can take up to several decades to become evident in populations. Surveillance of the workplace and general environmental monitoring are not being widely conducted at this time. The last national survey of workplace carcinogens was conducted in the last century, and no new survey is planned at this time. Furthermore, except in cases where the cancer risks are enormous, such as tobacco smoking and work-place exposure to asbestos, linking a chemical outside the workplace, for example, in the diet, air, or water, to human cancers is virtually impossible.

In fact, the 2.5- to 3-year bioassays of the European Ramazzini Foundation of Oncology and Environmental Sciences on toluene, benzene, radiation, and aspartame (Soffritti et al. 2004, 2007) consistently indicate that the results of 2-year studies—the normal length of rat studies—under-predict potency/carcinogenicity and that true lifetime studies more accurately reflect cumulative impacts.

In addition, the paradigm-busting work of researchers on transgenerational effects, male-mediated teratogenesis, and the general critical impact of early developmental windows all strengthen the case for regarding 2-year postnatal bioassays as incomplete indications of toxicity/carcinogenicity (e.g., Dolinoy et al. 2007; Newbold and McLachlan 1982; Sonne et al. 2008; Swan et al. 2006).

In our commentary (Huff et al. 2008) we indicated that 2-year results tend to be biased toward the null hypothesis. In no way do we state or imply that a positive finding in a 2-year assay would be negated by a longer test, nor do we indicate the contrary, that the absence of a positive finding in a 2-year assay should be construed as proof that there is no impact.

The current typical bioassay has served society well, but it does have several flaws: It cannot provide adequate prediction for the growing proportion of the population that is now living well into their eighties and nineties; it is not designed to evaluate the impact of prenatal exposures on later life; rodents are not a perfect model for humans; and countless animals must be sacrificed to obtain admittedly imperfect results.

By focusing improved bioassays on high-volume chemicals for which there are *a priori* grounds for concern, current approaches promise to yield the greatest good with the least harm. Simultaneously, more research must be done to identify fast, sensitive, accurate, and economical means of identifying chemicals that might be harmful to humans.

## References

[b1-ehp-116-a517] Dolinoy DC, Huang D, Jirtle RL (2007). Maternal nutrient supplementation counteracts bisphenol A-induced DNA hypomethylation in early development. Proc Natl Acad Sci USA.

[b2-ehp-116-a517] Huff J, Jacobson MF, Davis DL (2008). The limits of 2-year bioassay exposure regimens for identifying chemical carcinogens. Environ Health Perspect.

[b3-ehp-116-a517] Newbold RR, McLachlan JA (1982). Vaginal adenosis and adenocarcinoma in mice exposed prenatally or neonatally to diethylstilbestrol. Cancer Res.

[b4-ehp-116-a517] Soffritti M, Belpoggi F, Padovani M, Lauriola M, Degli Esposti D, Minardi F (2004). Life-time carcinogenicity bioassays of toluene given by stomach tube to Sprague-Dawley rats. Eur J Oncol.

[b5-ehp-116-a517] Soffritti M, Belpoggi F, Tibaldi E, Esposti DD, Lauriola M (2007). Life-span exposure to low doses of aspartame beginning during prenatal life increases cancer effects in rats. Environ Health Perspect.

[b6-ehp-116-a517] Sonne SB, Kristensen DM, Novotny GW, Olesen IA, Nielsen JE, Skakkebaek NE (2008). Testicular dys-genesis syndrome and the origin of carcinoma in situ testis. Int J Androl.

[b7-ehp-116-a517] Swan SH (2006). Prenatal phthalate exposure and anogenital distance in male infants. Environ Health Perspect.

